# Environmental-stress-induced Chromatin Regulation and its Heritability

**DOI:** 10.4172/2157-2518.1000156

**Published:** 2014-01-15

**Authors:** Lei Fang, Kenly Wuptra, Danqi Chen, Hongjie Li, Shau-Ku Huang, Chunyuan Jin, Kazunari K Yokoyama

**Affiliations:** 1 Department of Environmental Medicine, NYU School of Medicine, 57 Old Forge Road, Tuxedo, NY 10987, USA; 2 Center of Environmental Medicine, Graduate Institute of Medicine, Kaohsiung Medical University, 100 Shih-Chuan 1st Rd, San Ming District, Kaohsiung 807, Taiwan; 3 Division of Environmental Health and Occupational Medicine, National Health Research Institutes, 35 Keyan Rd, Zhunan, Miaoli County 350, Taiwan; 4 Johns Hopkins Asthma and Allergy Center, Johns Hopkins University School of Medicine, Baltimore, MD 21224, USA

**Keywords:** Chromatin assembly, Chromatin regulation, Endocrine-disrupting chemicals, Environmental stress, Epigenesis, Epigenetic inheritance

## Abstract

Chromatin is subject to proofreading and repair mechanisms during the process of DNA replication, as well as repair to maintain genetic and epigenetic information and genome stability. The dynamic structure of chromatin modulates various nuclear processes, including transcription and replication, by altering the accessibility of the DNA to regulatory factors. Structural changes in chromatin are affected by the chemical modification of histone proteins and DNA, remodeling of nucleosomes, incorporation of variant histones, noncoding RNAs, and nonhistone DNA-binding proteins. Phenotypic diversity and fidelity can be balanced by controlling stochastic switching of chromatin structure and dynamics in response to the environmental disruptors and endogenous stresses. The dynamic chromatin remodeling can, therefore, serve as a sensor, through which environmental and/or metabolic agents can alter gene expression, leading to global cellular changes involving multiple interactive networks. Furthermore its recent evidence also suggests that the epigenetic changes are heritable during the development. This review will discuss the environmental sensing system for chromatin regulation and genetic and epigenetic controls from developmental perspectives.

## Introduction

Although many genetic and epidemiologic studies have indicated that genetic variation strongly influences disease susceptibility, exposure to environmental factors clearly affects the onset, progression, and severity of germ-related and inflammatory diseases [1–3]. In fact, recent epidemiologic studies have identified a variety of risk factors for the development of many common diseases. Among them, air pollution, fuel exhaust, smoking, Polycyclic Aromatic Hydrocarbons (PAHs), volatile organic chemicals and environmental disrupting chemicals (EDCs) have been suggested as the culprit in triggering or exacerbating diseases [4–6]. The most commonly studied EDCs are dichlorodiphenyltrichloroethane (DDT), polychlorinated biphenyls (PCBs), polybrominated diphenyl esters (PBDEs), phthalates, and bisphenol A (BPA). This has been exemplified clearly by several recent meta-analyses of residential environmental chemicals and the occurrence primarily of allergic and immunological diseases [7]. These findings are particularly relevant when the increase in the prevalence of allergic and immune diseases in industrialized countries over the last decades is taken into consideration. One intriguing hypothesis is that the expression and increased prevalence of these diseases are attributable to these new immune “adjuvant” factors, e.g., environmental chemicals such as EDCs [8].

In the context of chemical carcinogenesis, direct damage of DNA by these environmental disruptors (or genotoxic agents) is the primary mechanism of chemical carcinogenesis. The maintenance of genome integrity in eukaryotes involves several damage-surveillance and DNA-repair enzymes. Similar to the gene-expression machinery, these enzymes must operate within the repressive chromatin environment of the nucleus [9,10]. DNA damage induced by genotoxic agents that modify DNA bases covalently or generate single-stranded DNA breaks are primarily recognized and repaired by DNA excision repair pathways [11]. In a manner that is analogous to the chromatin decomposition that accompanies the recruitment of RNA polymerase II during gene activation, DNA excision repair is associated with increased histone acetylation and localized chromatin remodeling [9]. Establishing the extent to which particular environmental disruptors influence genome function is an essential first step in defining their potential effects on the long-term viability of the target organism. If an environmental disruptor can induce an epigenetic change that is heritable through mitosis, and persists even in the absence of the original factor, there is a potential for significant phenotypic effects long after the initiating factor has disappeared. Furthermore, if such mitotically heritable changes are induced in germ cells, then the transmission through meiosis to succeeding generations might be possible. It is well known, through many years of work on imprinted genes, that epigenetic effects (i.e., whether a gene is expressed or not without the change in DNA sequences) can be transmitted through the germ line [12,13].

In this review, mechanisms via which environmental disruptors impact genome function through chromatin regulation in higher eukaryotes are discussed. We focus on molecular mechanisms that operate through families of enzymes that modify chromatin and chromatin-associated proteins. We also introduce recent advances in the study of environmental toxicant-mediated dysregulation of chromatin assembly, and discuss how an environmental agent might induce a change in gene expression that is heritable through mitosis (epigenetic mutation), or even through the germ line, to subsequent generations.

## Chromatin Landscapes

Epigenetic modifications in response to environmental disruptors include histone modifications, DNA methylation, ATP-dependent chromatin remodeling, and dysregulated microRNAs [14]. The histone modifications were separated into distinctive clusters, indicating their relations to different genome features. For example, modifications associated with active promoters (H3K4me3 and H3K9ac), transcribed regions (H3K36me3), and distal elements (H3K4me1 and H3K27ac) were correlated with one another but showed varying degrees of exclusivity, with repressive marks for H3K27me3 and H3K9me3 [9]. It is becoming increasingly clear that a variety of environmental factors change gene expression through shifting covalent histone modification profiles. For example, exposure to BPA, an endocrine disruptor, in human breast cancer MCF7 cells and mammary glands of six-week-old mice, has been shown to increase the levels of H3K27me3, which is associated with gene silencing [15]. Most carcinogenic metals change genome function by epigenetic mechanisms. A nontoxic dose of nickel is shown to increase global levels of H3K4me3 and H3K9me2 [16]. Moreover, acute nickel exposure increases levels of H3K9me1 and H3K9me2 in several cell lines [17]. Recently it has been demonstrated that nickel exposure alters global levels of H3K4me3 and H3K9me2 in nickel-exposed refinery workers [18]. These modifications are catalyzed by chromatin-modifying enzymes.

Environmental factors appeared to alter the histone modifications at least in part by directly regulating the levels and/or activities of histone modifying enzymes. For example, hypoxia and exposure to nickel increased the level of H3K9me2 by inhibiting histone demethylase JMJD1A [19]. Moreover, hypoxia and hypoxic mimetic increased the protein and enzymatic activity of G9a, a H3K9 methyltransferase [20]. Interestingly, chromatin-modifying enzymes are generally susceptible to the cellular microenvironment, i.e., the levels of various metabolites. The NAD-dependent class III deacetylase SIRT1 acts on both histones and transcription factors, such as p53 [21] and the androgen receptor [22], thus providing an interesting link with the cellular metabolic state of the cell [23,24]. A high NAD/NADH ratio enhances SIRT1 activity, with deacetylation of the androgen receptor and diminution of its growth-promoting activity. Conversely, low levels of NAD, or high levels of the inhibitor (and SIRT product) nicotinamide suppress SIRT1 and may act as a sensor of the redox state of the cells [25].

5-Methy-cytosine (5meC) represents 2–5% of all cytosines in mammalian genomes and is found primarily on CpG dinucleotides [26]. DNA methylation regulates many cellular processes including chromatin structure remodeling, X-chromosome inactivation, genome imprinting, chromosome stability, and gene transcription [12,27]. In general, hypermethylation of the promoter of a gene is associated with decreased expression of that gene [28]. Conversely, hypomethylaiton associated with a noncoding region has been linked to chromosome instability [29]. Alterations of DNA methylation patterns, such as global hypomethylation and hypermethylation of CpG islands in promoter regions, have been found increasingly in different types of tumors. Exposure to a variety of environmental disruptors can alter DNA-methylation patterns. For example, oxidative stress has been associated not only with global hypomethylation, but also with increasing dense methylation of specific genes [30]. These changes could result in aberrant genome stability and gene expression, thereby leading to cell transformation and tumorigenesis. DNA methylation may also contribute to environmental tumorigenesis through dysregulating microRNA expression, since the expression of microRNAs are also reported to involve DNA methylation mediated by exposure to environmental disruptors, such as dichlorvos (DIC) [31], conazoles [32], and arsenic [33].

In addition to histone modifications and DNA methylation, chromatin could be remodeled through ATP-dependent chromatin remodeling factors to either activate or silence a gene. Nucleosome positions regulate DNA accessibilities and play important roles in DNA-dependent processes. ATP-coupled chromatin remodeling complexes controls nucleosome positions by translocating nucleosomes or evicting them from the DNA. A number of studies have shown roles of ATP-dependent chromatin remodeling factors in responding to environmental stresses. SWI3B [Imitation of SW (Switch) 3B] is a subunit of *Arabidopsis* SWI/Sucrose Non Fermentable (SNF) complexes. *Swi3b* mutants abolished the presence of a key regulator of anscisic acid (ABA) signaling in the vicinity of ABA-response genes, and reduced their expression [34]. PICKLE (PKL) is the chromodomain and helicase-like domain (CHD) ATPase in *Arabidopsis*, which inhibits expression of some ABA-response genes. Perruc et al. [35] reported that *pkl* mutants reduced the levels of histone repressive marks at promoters of some genes upon ABA treatment, indicating that PKL is necessary to maintain chromatin of these genes in a repressed state. There are a number of different types of remodeling complexes in mammalian cells with specific functions. These complexes translate a variety of signals into certain patterns of nucleosome positions. Environmental genotoxins cause a variety of DNA lesions, which if not repaired properly, can lead to cancer. Growing evidence has suggested that ATP-dependent chromatin remodeling factors play important roles in the DNA-damage response. Many different types of remodelers including ISWI and WICH [WSTF (the Williams syndrome transcription-factor)/ISWI chromatin remodeling] complex are recruited to DNA damage sites upon genotoxic exposure [36,37]. ISWI complexes influence access of repairing factors to DNA by translocating nucleosomes. Moreover, they also serve as a docking or signaling site for repair and signaling proteins. For example, ISWI subunit Acf1 recruited Ku70/80 complex to the repair site, and WICH subunit WSTF protein phosphorylated H2A.X, which served as a signal for downstream reactions [38]. These studies indicate significance of ATP-dependent remodeling factors in regulating chromatin structure in responding to different environmental cues, while the molecular mechanisms involved in the process need further investigation. It would be interesting to examine if environmental disruptors modulate chromatin structure by directly targeting ATP-dependent chromatin remodelers.

## Chromatin Assembly

Epigenetic mechanisms give rise to different patterns of gene expression and define cell fate in multicellular organisms. Growing evidence shows that environmental factors can change epigenetic profiles through covalent chromatin modifications, such as DNA methylation and posttranslational histone modifications, thereby changing gene expression and cellular phenotype. In addition to chromatin modifications, proper assembly and disassembly of chromatin itself are also crucial, as they ensure the maintenance of epigenetic information, and control DNA accessibility, genome instability, and transcription. Nucleosomes can be assembled in a replication-coupled or replication-independent manner. Canonical histone H3 (H3.1 and H3.2 in mammals) is assembled into chromatin solely during S phase, after DNA replication via a replication-coupled mechanism [39,40]. However, variant histone H3.3, which differs from canonical H3 by four or five amino acids, is deposited throughout the cell cycle in a replication-independent manner [39,40]. Histone chaperones bind to histones and regulate histone dynamics, such as transfer, transport, or storage, thereby modulating chromatin assembly [41]. Thus, histone deposition is assisted by chaperone proteins. For example, canonical histone H3 is incorporated into chromatin by chromatin assembly factor 1 (CAF-1) during DNA replication [42], whereas several histone chaperones, such as HIRA [43], death-domain associated protein (DAXX), α-thalassaemia/mental retardation syndrome X-linked (ATRX), and DEK, are responsible for the delivery of variant histone H3.3 into different genomic loci [44–47]. Among them, HIRA mediates H3.3 loading onto genetic regions and some regulatory regions, and DAXX directs H3.3 deposition at regulatory regions, and DAXX and ATRX are tightly associated with H3.3, and ATRX targets DAXX to telomeres and pericentric heterochromatin in MEFs. In *Drosophila*, dDEK is found to be preferentially associated with histones enriched with active epigenetic marks, and facilitates H3.3 assembly during puff formation.

In addition to histone chaperones, appropriate covalent modifications of nascent histones H3 and H4 play critical roles in histone nuclear import and assembly into chromatin. Newly synthesized histones H3 and H4 are acetylated before being assembled into chromatin. H4K5 and H4K12 are acetylated by the type B histone acetyltransferase Hat1. H4K5 and H4K12 acetylation regulates the interaction between H3/H4 and importin 4, which is a nuclear transport receptor, and another chaperone anti-silencing function 1 (ASF1) [48–50]. Mutations in H4K5 and H4K12 lead to impaired nuclear translocation compared with wild-type histones [51,52]. Moreover, histone H4 associated with the Hat1 complex is noted to harbor acetylation at the K91 residue located in its globular domain [53]. Mutation at this site yields phenotype changes, such as sensitivity to DNA damage and derepression of silent chromatin, when chromatin assembly is defective [53]. In yeast, acetylation of five lysine residues at H3 (K9, K14, K23 and K27) and the modifying enzyme, Gcn5, facilitate nucleosome assembly [50]. In *Drosophila*, truncation mutation of the N-terminal tail of H3 compromised replication-coupled nucleosome assembly, indicating that the role of the N-terminal tail of histone H3 in acetylation appears to be conserved among different species [39]. Moreover, H3K56 acetylation is important for nucleosome assembly during DNA replication and repair, both in budding yeast and humans, at least in part by enhancing the interaction between CAF-1 and H3/H4 [51,52,54–56].

## Alteration of Chromatin Assembly by EDCs

Abnormal chromatin assembly may be a cause of, or a significant contributor to the pathogenesis of various diseases, such as cancer [57]. Most direct evidence has come from recent deep-sequencing that showed mutations in the DAXX–ATRX–H3.3 pathway in several cancers [58–63]. Somatic mutations in the H3.3–ATRX–DAXX chromatin-assembly pathway have been identified in 44% of pediatric glioblastoma tumors, suggesting that these mutations might be “driver” mutations for this type of cancer [60]. Moreover, 43% of pancreatic neuroendocrine tumors (PanNETs) had mutations in genes encoding ATRX and DAXX [62]. ATRX/DAXX is important for H3.3 deposition at telomeres. Not surprisingly, PanNETs containing ATRX/DAXX mutations exhibit abnormal telomeres. A DAXX missense mutation was also found in acute myeloid leukemia [64]. Given the role of DAXX in PML-RARα-driven transformation [65], aberrant H3.3 deposition may also play a role in the pathogenesis of leukemia. Overexpression of another histone H3.3 chaperone, DEK, has been detected in several cancers, which facilitates epithelial transformation [66]. In some human myeloid leukemia patients, DEK is fused to CAN by chromosomal translocation and its mutation is found to significantly reduce the formation of the DEK complex and H3.3 loading [46,67]

Although it is becoming increasingly apparent that chromatin assembly is directly associated with cancer initiation and development, little is known about the effects of the environment on chromatin-assembly pathways. In plants, different abiotic stresses are known to downregulate the expression of histone chaperones, suggesting that aberrant nucleosome assembly is involved in the regulation of stress responses, although the mechanisms underlying this process have not been clarified [68]. Whether and how environmental factors influence the posttranslational modifications (PTMs) of newly synthesized histones and histone chaperone proteins, thereby leading to changes in nuclear import and chromatin assembly, remains largely unknown.

**Figure 1:**
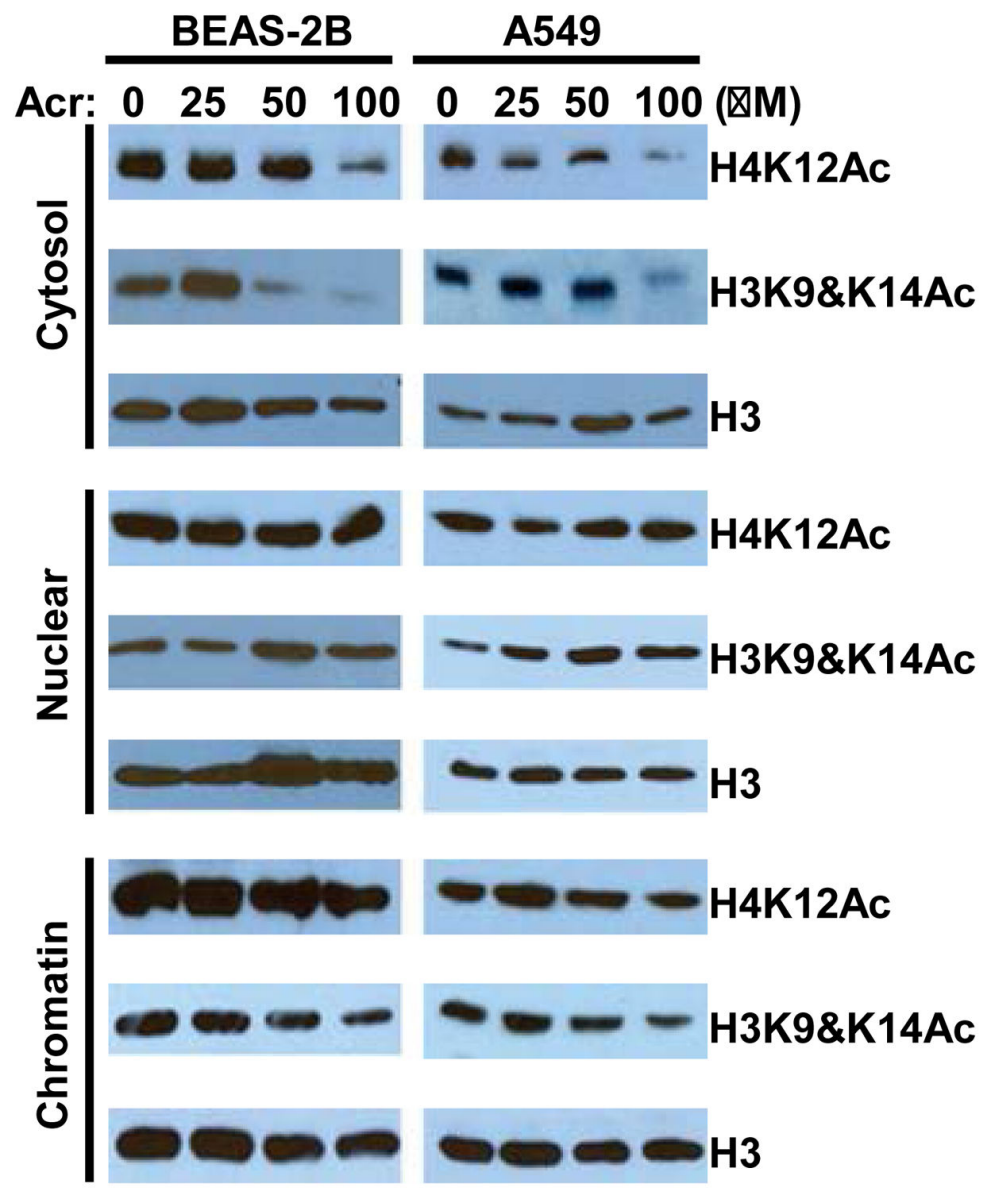
Acrolein inhibits N-terminal tail acetylation of newly synthesized histones. Cytosolic cell fractions, nuclear extracts, and soluble chromatin fractions were isolated from BEAS-2B or A549 cells treated with or without Acrolein (Acr) for 2 h and then subjected to Western blot. The results showed drastic decrease of cytosolic H4K12Ac and H3K9 and K14Ac by Acr exposure [91].

In an attempt to understand the epigenetic mechanisms that control acrolein (Acr) pathogenesis, we recently found that interfering with chromatin assembly may represent a major mechanism via which Acr functions. Acr is an α,β-unsaturated aldehyde that is abundant in the environment. It is derived from automobile exhaust and industrial emissions and is enriched in tobacco smoke and heated cooking oil [69–72]. Acr has been implicated in the development of multiple sclerosis, Alzheimer’s disease, pulmonary disorders, cardiovascular diseases, among others [69,72–84]. Specifically, Acr has been noted as a potential major carcinogen associated with smoking-related lung cancer [70,78]. While PAHs have long been considered the major carcinogens in tobacco smoke [85], because they are linked to p53 mutational patterns in lung cancer. This notion has recently been challenged by our finding that Acr, which is 1000-fold more abundant than PAHs in tobacco smoke, also binds preferentially to p53 mutational hotspots [70]. As a highly reactive aldehyde, Acr not only reacts with nucleophilic guanine bases in DNA [24,70,78,85–87], but, in theory, it may target multiple amino acid residues, including lysine, within susceptible proteins [69,88]. By using antibodies against cyclic lysine adducts, several studies reported the formation of Acr–lysine adducts *in vivo* in the affected tissues of individuals with several degenerative diseases [89]. As histone proteins are rich in lysines, it raises the possibility that Acr also targets lysine residues in histone tails, thereby affecting their PTMs. Because appropriate histone lysine modifications are crucial for genome function [90], the formation of Acr-histone adducts is expected to have a significant impact on cellular processes. In fact, we have shown that Acr reacts with histone proteins and specifically downregulates the acetylation of the N-terminal tails of cytosolic histones H3 and H4 [91] (Figure 1). The reduction of histone acetylation appears not to be because of the changes in histone acetyl transferase (HAT) expression and/or activity, as the expression of HAT1, which is specific for H4K5 and H4K12 acetylation, was not changed by Acr exposure, and also the *in vitro* total HAT activity of cytosolic fractions from Acr-treated cells was not altered compared with that from wild-type cells (data not shown). As mentioned above, appropriate acetylation of the N-terminal tails of newly synthesized histones H3 and H4 is believed to be important for histone nuclear import and nucleosome assembly [48,51,52,54,55,92–94]. In fact, Acr exposure led to a compromise of chromatin assembly. This conclusion was supported by the following results. The levels of histone H3 marked with H3K9 and K14 acetylation were drastically decreased after Acr exposure at the majority of genomic loci tested. The results seemed not to be due to a direct reaction of Acr with nucleosomal histones, because H3K4me3, which is another active mark, was not downregulated in parallel. Moreover, while γ-tubulin levels were not changed, the amount of H3 in chromatin fractions was depleted by Acr exposure. Importantly, Acr treatment facilitated the accessibility of chromatin, as evidenced by partial MNase digestion assays. In the future, it would be interesting to test whether Acr exposure disrupts the associations of H3/H4 with nuclear import and histone chaperone proteins. Acr may also target histone chaperones, thereby changing the chromatin assembly pathway [95]. Although the expressions of the CAF-1 p150 subunit, importin 4, and ASF1 were not affected by Acr exposure (data not shown), whether Acr may modify these proteins and affect their functions need to be determined.

Because of their similar chemical properties, we further determined whether other aldehydes, such as formaldehyde and acetaldehyde, also react with nascent histones and influence chromatin assembly. We found that both formaldehyde and acetaldehyde specifically reduce the acetylation of cytosolic histones and compromise histone delivery similar to Acr (data not shown). Thus, the regulation of chromatin assembly appears to be a common characteristic of aldehydes. Other electrophiles, such as metabolically activated PAHs and alkylating agents, may also have similar effects. Moreover, Acr can be generated endogenously by oxidative stress, which occurs often in cells exposed to environmental agents [69,71,72]. Thus, reaction with free histones, which regulate the thereby regulating nucleosome-assembly pathway, may represent an important mechanism via which a wide range of environmental factors interact with the genome and influence genome functions.

## Heritable Implication of Cell Memory and Germ line

**Figure 2:**
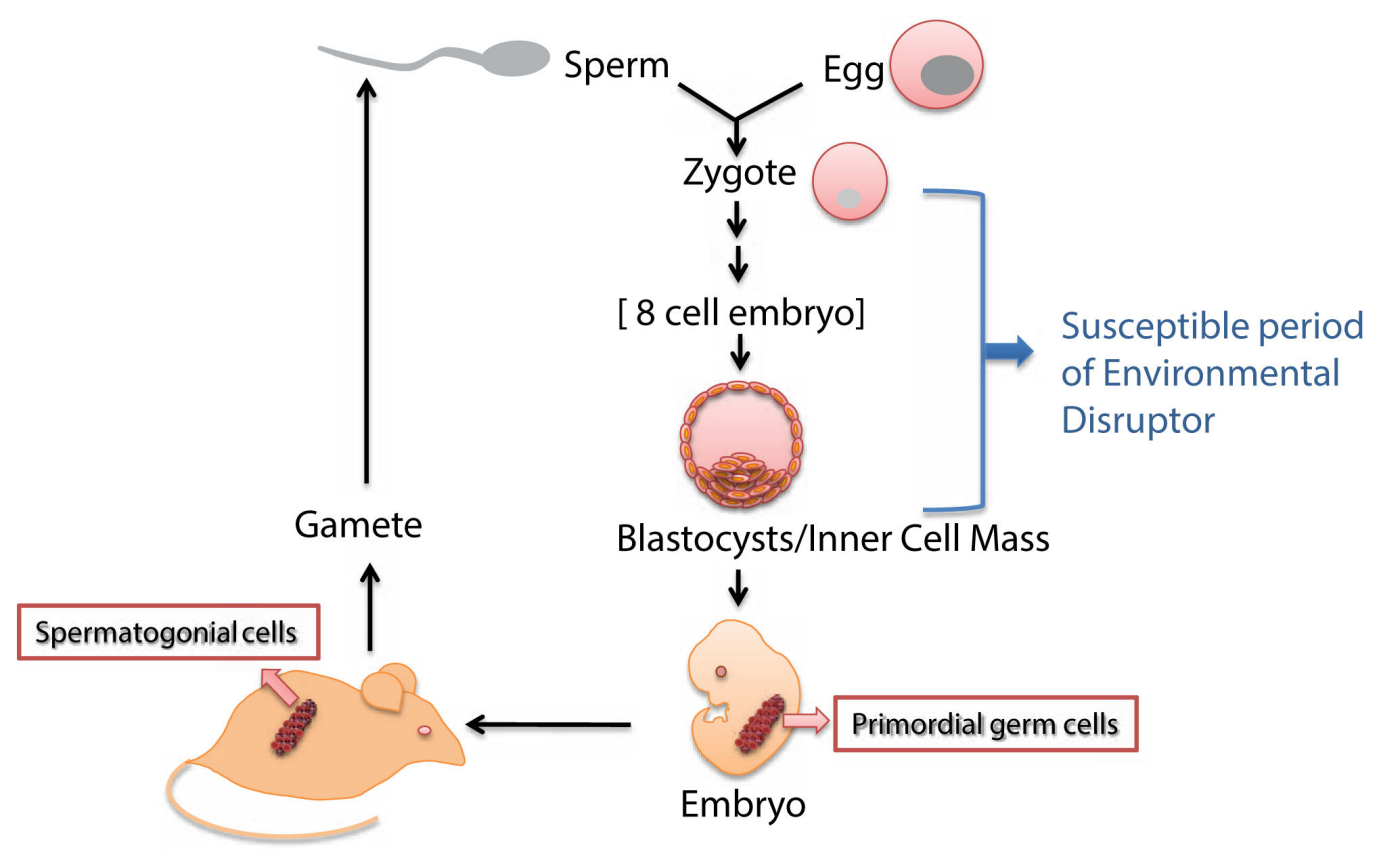
Schematic representation of heritable epigenetic change in the early embryo. The susceptible period is the developmental stage at which changes in histone modification levels, perhaps at only some genes, induced by environmental disruptors, can be passed on, through mitosis, to subsequent cell generations. If induced changes are transmitted to the primordial germ cells (PGCs) and hence into the gametes, they could be transmitted through the germ line to the next generation.

If mitotically-heritable epigenetic changes are induced in germ cells, then there is a possibility for transmission of epigenetic changes through meiosis to the succeeding generations. Imprinting genes are well known in this category, although the underlying mechanisms remain unclear [12]. DNA methylation is likely to be involved, but seems not to provide a complete explanation. Attempts to demonstrate experimentally the germ-line inheritance of induced phenotypic changes have met with difficulties. Transmission through the male germ line presents particular problems for epigenetic inheritance. During fertilization, sperm DNA is repackaged with maternal histones, followed by an overall loss of methylated cytosine. However, it is likely that some regions, such as imprinted genes, are protected from demethylation [96], while careful analyses have shown retention of a small amount of histone in sperm chromatin [97], with enrichment of selected variants, such as H3.3 and H2A.Z [98]. Thus, sperm histones are associated with specific genes, perhaps with those that need to be expressed very early in zygotic development [99]. Although we acknowledge the complexity and experimental challenges of research on epigenetic inheritance, further efforts are certainly required. If environmental disruptors can induce a heritable change in, for example, histone modification in somatic cells, then it is likely that this can also happen in germ cell precursors and be transmitted to the germ-cells themselves and, thence, to the zygote. Effects on the developmental embryo and the adult organism will depend on the genes involved, but still it remains unclear. Figure 2 summarizes how a mitotically-inheritable epigenetic change, induced during the preimplantation stage of embryogenesis, can be transmitted to germ cells and to the next generation. The most sensitive period to the environmental stresses in the epigenetic inheritance of the early embryonic development might be between the 8-cell morula embryo stage and the blastocyst stage, based on studies of the HDAC inhibitor valproic acid [100]. The same environmental disruptors will usually cause selective epigenetic changes at multiple loci or gene clusters and, especially, both alleles of each susceptible gene will be affected equally.

In primordial germ cells (PGCs), there is extensive erasure of epigenetic marks [101,102]. The newly induced epigenetic change must survive this if it is to be passed to the next generation. Currently it is impossible to say how frequently this may occur. However, the epigenetic marks that determine the zygotic expression of imprinted genes depending on whether they have been passed through the maternal or paternal germ line, survive this erasure process. Moreover, there is extensive demethylation of DNA in the zygote and preimplantation embryo [103], which may erase some epigenetic marks. However, this erasure should not be complete and even marks that are dependent on DNA methylation may survive. Then, this epigenetic inheritance will be transmitted to the next generation. The current model involves an epigenetic transgenerational inheritance of a behavioral phenotype induced by environmental disruptors [104,105] that is transmitted through the germ line and involves a permanent alteration in the sperm epigenome (i.e., DNA methylation). The epigenetic transgenerational inheritance of this altered sperm epigenome modifies the subsequent development and epigenomes of all cells and tissues, including the brain, to promote phenotypic variation [106]. Although no direct epigenetic measurements were made in the current study, the epigenetic model and role of epigenetics in development provide the molecular basis of the observations presented [107].

## Speculation Regarding the Events that Link Environmental Factors to Genomic Change

**Figure 3:**
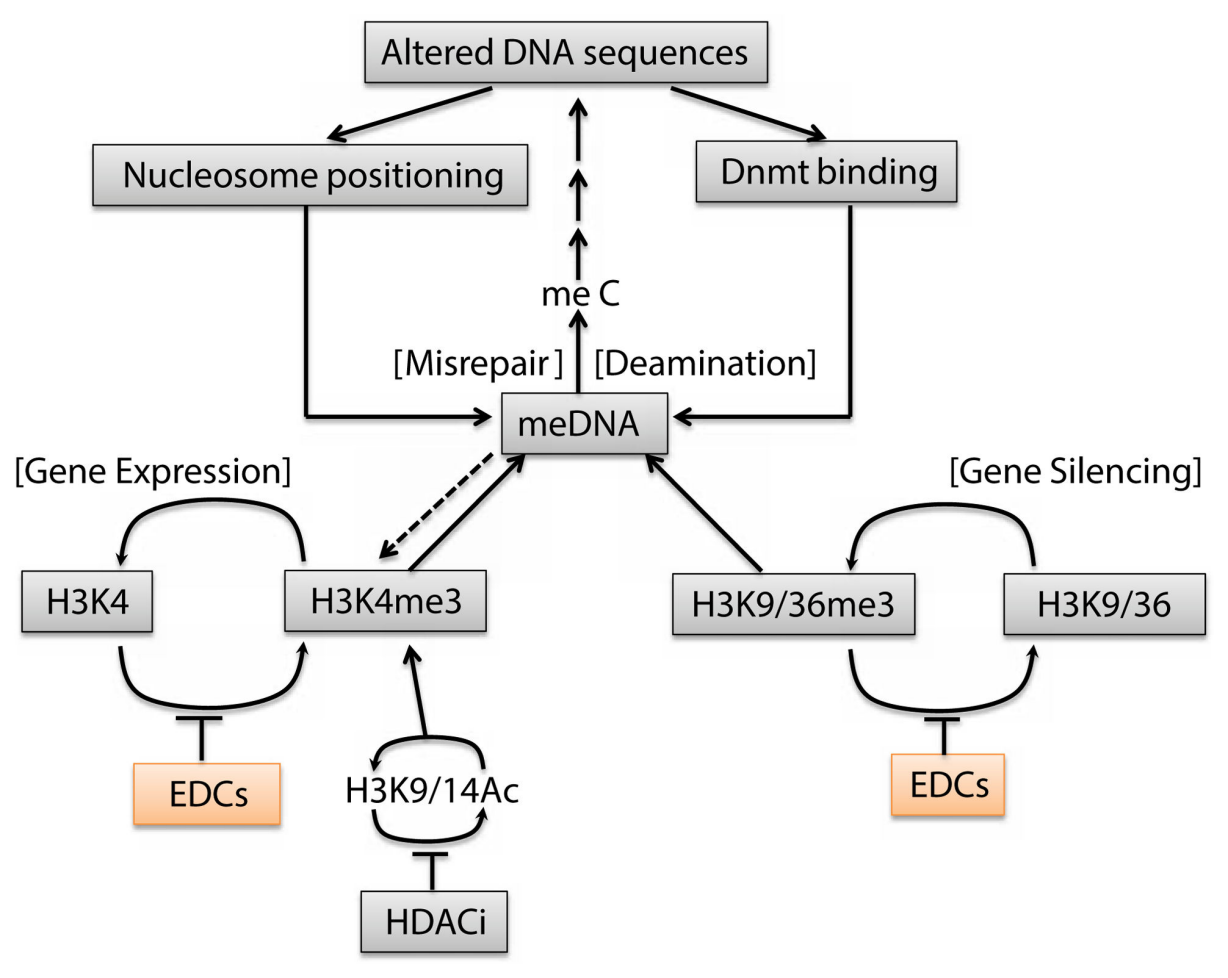
How an environmentally-induced epigenetic changes might alter DNA sequence. The chain of events shown is speculative but the individual elements are all based on established mechanisms. The process starts with inhibition of demethylases of H3K9me3 and H3K37me3 as well as methylase of H3K4me3 in chromatin by environmental disrupting chemicals (EDCs). These results increase in H3K9me3, H3K36me3 and HeKme3, which may be global or local depending on the distributing enzymes. Then, H3K9me3 and H3K4me3 can modulate to activate or repress DNA methyltransferases (Dnmt), leading to increase or decrease of DNA methylation. In higher eukaryotes, DNA methylation occurs at the cytosine of CpG dimers. The slow, but inevitable, deamination of 5′ methyl cytosine (meC) forms thymidine (T), resulting in a G-T base mismatch, repair of which involve replacement of either base. Replacement of the G with an A results in an altered DNA sequence on both strands, in which the original meC is replaced with T. Such a change could exert phenotypic changes, even if it does not occur in a coding region or transcription factor binding sites. Both nucleosome positioning and binding of DNA methyltransferases and DNA demethylase are known to be dependent on DNA sequences, through the sequences involved are complex. Histone deacetylase inhibitor (HDACi) is also affective to the state of acetylation of H3K9 or H3K14, which causes to stimulate the trimethylation of H3K4. Over evolutionary time, localized changes in DNA sequences might result eventually, in a region of silencing or activating determined genetically by DNA sequence, rather than epigenetically.

A possible flow chart of how an environmentally-induced epigenetic change might alter a DNA sequence is summarized in Figure 3. High levels of H3K4 methylation protect DNA in chromatin from cytosine methylation at CpG dimmers, and the levels of H3K4 methylation are influenced by other H3 modifications, including acetylation, exerting an indirect effect on DNA methylation. In some organisms, the methylation of H3 at K9 or K36 can also influence the levels or sites of DNA methylation. Metabolic or environmental components that shift the equilibrium that is maintained by one or more of these enzyme cycles, can potentially alter the local levels of DNA methylation. In higher eukaryotes, DNA methylation occurs at the cytosine(s) of CpG dimers. The slow, but inevitable, deamination of 5′-methyl cytosine (meC) from thymidine (T), results in a G–T base mismatch, the repair of which may involve the replacement of either base. The deamination of cytosine forms uracil, which is invariably replaced. Replacement of the G with an A results in an altered DNA sequence on both strands, in which the original meC is replaced with T. Such a change might have phenotypic effects, even if it does not occur in a coding region or at transcription factor binding sites. Both nucleosome positioning and binding of DNA methyltranferases are dependent on DNA sequence, although the sequences involved are complex. Over evolutionary time, localized changes in DNA sequences, perhaps through their effects on nucleosome positioning and Dnmt binding, might result, eventually, in a region of silencing that is determined genetically by DNA sequence, rather than epigenetically, as originally proposed [108].

## Conclusions

The idea that acquired traits induced by environmental conditions may become heritable dates back to Lamarck and has been controversial ever since [109]. Over the past century, many studies have attempted to demonstrate that parental environment can influence directly the phenotype and fitness of the offspring; however, it is still not conclusive in the animal world. The activity of chromatin modifying enzymes can be influenced by a range of metabolites whose levels can be influenced by diet and lifestyle, by a variety of environmental chemicals, and by climatic changes, amongst other factors. The changes induced can be widespread, affecting all or most members of a population, and rapid, occurring in parallel with exposure to the inducing agent. It may also be significant that epigenetic changes would be able to regularly affect multiple, possibly functionally related, genes and gene families, depending on how the enzymes involved are targeted. The persistence of the environmental agent that induces the epigenetic changes would give natural selection time to act on the altered phenotype without a need for the heritability of the induced changes. All these characteristics argue that induced epigenetic changes can make a significant contribution to evolution.

The response of any individual organism to an environmental disruptor will depend on the families of chromatin-modifying enzymes involved and their ability to metabolize or otherwise deal with the disruptors. Genetic polymorphism generated by a combination of genetic and epigenetic mechanisms may have selectable phenotypic consequences. The question remaining regards the determination of the extent to which environmentally induced epigenetic changes can contribute to the pool of variants on which natural selection operates. Such mechanisms not only have long-term implications for evolutionary changes themselves, but are of immediate relevance to human and animal health.
